# Bioaccessible Bread Melanoidins Modulate Oxidative Stress, Reduce Inflammation and Suppress Adhesion of *Helicobacter pylori* to Caco-2 Cells

**DOI:** 10.3390/nu17040648

**Published:** 2025-02-11

**Authors:** Gisela Gerardi, Virginia Temiño, Gonzalo Salazar-Mardones, Noelia Díaz-Morales, Beatriz Melero, Carolina Bocigas, Pilar Muñiz, Jordi Rovira, Mónica Cavia-Saiz

**Affiliations:** Department of Biotechnology and Food Science, Faculty of Sciences, University of Burgos, Plaza Misael Bañuelos, 09001 Burgos, Spain; vtemino@ubu.es (V.T.); gsalazar@ubu.es (G.S.-M.); ndiaz@ubu.es (N.D.-M.); bmelero@ubu.es (B.M.); cbocigas@ubu.es (C.B.); pmuniz@ubu.es (P.M.); jrovira@ubu.es (J.R.); monicacs@ubu.es (M.C.-S.)

**Keywords:** melanoidin, crust bread, *H. pylori*, antimicrobial, adhesion, oxidative stress, inflammation

## Abstract

**Background/Objectives**: *Helicobacter pylori* is a major contributor to gastric infections; it is prevalent in humans and associated with gastrointestinal diseases. In recent years, the increase in antimicrobial resistance has contributed to the need for alternative approaches, prompting interest in natural products with antimicrobial and antivirulence properties. This study investigated the effect of bioaccessible melanoidins from common and soft bread crust against *H. pylori* infection. **Methods**: Melanoidins were extracted using dead-end ultrafiltration, and bioaccessible fractions were obtained through in vitro digestion. The bactericidal effect of melanoidins was assessed at 2% and 4% concentrations over 24 and 48 h. The effect on *H. pylori* adhesion of 100 μg/mL and 200 μg/mL of gastric and intestinal bioaccessible fractions of melanoidins was evaluated in Caco-2 cells. **Results**: The bactericidal effect of melanoidins revealed significant efficacy, with a greater effect for soft bread melanoidins. The gastric fractions exhibited a higher inhibitory effect, which is crucial for gastric mucosa, the primary site of *H. pylori* infection. Both bioaccessible fractions showed anti-inflammatory and antioxidant effects against *H. pylori*-induced inflammation, particularly in the gastric fractions. This was evidenced by a reduction in interleukin-6 and interleukin-8 release and an enhancement in interleukin-10 release. The observed reduction in reactive oxygen species (ROS) and the maintenance of glutathione levels indicate an improved redox status. **Conclusions**: This study emphasizes the potential of melanoidins, especially from soft bread, as bioactive compounds against *H. pylori*, offering insights for future functional food development.

## 1. Introduction

*Helicobacter pylori* is a Gram-negative pathogen capable of colonizing and invading the gastrointestinal epithelium, and it is the primary cause of gastric complications, such as gastritis, peptic ulcer, and gastric cancer [1,2]. A large proportion of the world’s population is infected with this bacterium, ranging from 20 to 50% in industrialized countries [3,4]. According to some authors, the *H. pylori* infection results in chronic inflammation and increased oxidative stress [5]. Additionally, other studies suggest that the infection with *H. pylori* induces the formation of specific pro-inflammatory and virulence factors that contribute to the survival of *H. pylori*, including those that modulate nuclear factor kappa B (NF-κB) activity [1]. The transcription factor NF-κB regulates the genes involved in cell proliferation, inflammation, and apoptosis, and it is activated in stress-related conditions like *H. pylori* infection. Furthermore, *H. pylori* infection induces oxidative stress and the recruitment of inflammatory cells, identified as playing a significant role in the promotion of gastric cancer [5,6].

The interleukin 8 (IL-8) is a pro-inflammatory cytokine secreted by gastrointestinal cells due to the presence of *H. pylori*, and it has a direct association with the development of gastric carcinoma [7]. Several studies indicate that the presence of IL-8 increases the transcriptional activity of NF-κB and that Akt induces adhesion molecules in gastric cancer cells. The activation of these signaling pathways is part of the cell transformation, proliferation, invasion, survival, angiogenesis, metastasis, and inflammation in cancer [8]. Levels of interleukin 6 (IL-6) are also elevated in *H. pylori*-positive individuals with gastric carcinoma [7]. Other authors indicate that the signal transducer and activator of transcription 3 (STAT3) and NF-κB regulate the production of many cytokines, such as IL-6 and IL-8, among others, and the expression of anti-apoptotic proteins; moreover, IL-6 is implicated in the phosphorylation of STAT3 and its activation [9]. In contrast, the anti-inflammatory cytokine interleukin 10 (IL-10), which is activated in response to inflammatory processes, is reduced during *H. pylori* infection, and this triggers a decrease in the immune response in the affected cells [1]. Moreover, low levels of IL-10 are associated with increased gastrointestinal inflammation intensity [7].

On the other hand, in response to the presence of *H. pylori*, there is an increase in the reactive oxygen species (ROS) in host cells, such as neutrophils and epithelial cells. Inside these cells, ROS are produced to eliminate the pathogenic bacteria with the nicotinamide adenine dinucleotide phosphate (NADPH) oxidase system. However, the problem is that *H. pylori* colonizes the light; so, it can evade these mechanisms. Consequently, the cells generate chronic infection that produces inflammation, oxidative stress, and damage to the gastric mucosa [1,10].

Melanoidins are the final products of the Maillard reaction (MR), generated by non-enzymatic reactions between protein amino groups and reducing sugars. Bread crust is an example of a food product rich in melanoidins, with data indicating that the daily intake of bread melanoidins ranges from 1.8 to 1.5 g per day [11]. Several studies suggest that melanoidins play a crucial role in biological activities with clear health benefits [12,13,14,15].

Melanoidins from bread crust have been shown to have antioxidant and antimicrobial activities, among others [12,16,17]. The antimicrobial activity of the melanoidins is related to different mechanisms associated with their metal-chelating properties; for example, the chelation of iron can produce bacteriostatic activity, and the chelation of siderophoro-Fe^+3^ complex can reduce the virulence of pathogenic bacteria [16,18]. It was suggested that melanoidins possess prebiotic activity as data from in vitro and in vivo studies showed that melanoidins suppressed *H. pylori* infection [19]. To exert their biological effects, melanoidins need to be released from the food matrix and absorbed across the intestinal barrier. In this regard, the digestion of melanoidins in the gastrointestinal tract may lead to the release of beneficial compounds that can be absorbed or exert their biological effects at the site. Therefore, our previous studies have focused on characterizing the melanoidins potentially serving as functional ingredients and their bioaccessibility [20,21].

Although melanoidins from bread crust have demonstrated antimicrobial activity [12,16,17] or anticolonization effects [15,19], there are limited studies on their efficacy against *H. pylori* cell infection. Moreover, the antioxidant capacity of melanoidins could play an important role in the modulation of inflammation and oxidative stress associated with *H. pylori* infection. In this study, we investigated their efficacy against the growth and adhesion of *H. pylori*; in addition, their capacity to modulate the inflammatory and oxidative stress in infected cells was studied.

## 2. Materials and Methods

### 2.1. Reagents

2′,7′-dichlorofluorescein diacetate (DCFH-DA), bovine serum albumin, fetal bovine serum, 200 mM L-Glutamine solution, Eagle’s minimum essential medium (MEM), 100X MEM non-essential amino acid solution, p-formaldehyde, phosphate buffer saline 1X (PBS), and Pronase E solution were purchased from Sigma-Aldrich, Co. (St. Louis, MO, USA). Enzyme-linked immunoabsorbent assay (ELISA) kits for interleukin 6 (IL-6), interleukin 8 (IL-8), and interleukin 10 (IL-10) were purchased from Cusabio Technology LLC (Houston, TX, USA). Recombinant anti-*Helicobacter pylori* antibody and goat anti-rabbit IgG H&L (Alexa Fluor^®^ 488; Abcam, Cambridge, UK) were purchased from Abcam (Cambridge, UK). Defibrinated horse blood was obtained from Thermo Fisher Scientific (Waltham, MA, USA). Columbia blood agar base and *Helicobacter pylori* selective supplement were purchased from Oxoid Ltd. (Hampshire, UK).

### 2.2. Bread Samples and Melanoidins Extraction

The extraction of melanoidins from common and sliced bread, along with their physicochemical characterization and cytotoxicity, has previously been described in studies [12,16,20]. Briefly, the crusts of bread products (soft bread and common bread) were used in this study. Commercial soft bread was obtained as byproducts from Cerealto S.A. factory (Palencia, Spain), and common bread was prepared in the pilot plant of the Food Technology Department of the University of Burgos. Common bread was prepared from 1 kg of wheat flour, 20 g of salt, 28 g of fresh yeast, and 500 mL of water. All the ingredients were first mixed in a kitchen mixer (KitchenAid, KSM90, Benton Harbor, MI, USA) and then manually kneaded. The dough was left to develop for 40 min at room temperature and placed in a fermentation cabinet (Salva Industrial SA, Guipuzcoa, Spain) for 30 min at 28 °C in high humidity. After the first fermentation, the dough was punched down, folded to the final shape, and placed again in the fermentation cabinet for 90 min. The proofed dough was baked in an electrical convection oven (Berto’s, Padova, Italy) at 220 ± 5 °C for 20 min to obtain common bread rolls. The separation of the crust from the attached crumbs in the common bread rolls and the soft bread byproducts was conducted by scraping their surfaces with a kitchen knife; thus, the brown-colored part (crust) was obtained, and the white crumb was discarded. Then, the obtained samples were ground in a coffee mill (Taurus Minimoka GR20, Taurus Group, Oliana, Spain) and sieved to a particle size of <1 mm with a 1 mm wire mesh sieve (CISA, Barcelona, Spain).

Subsequently, the melanoidins were extracted with Pronase E according to the method described by Roncero-Ramos et al. (2013) [15], with slight modifications. Briefly, 125 g of crust powder was enzymatically hydrolyzed with 750 mL of a 400 U/mL solution of Pronase E in 20 mM Tris-HCl buffer (pH 8.0) for 72 h at 37 °C with continuous agitation in the digestion jars of a DaisyII incubator (ANKOM Technology Corporation, Macedon, NY, USA). The in vitro digestion reactions were stopped by mixing the samples with 15 mL of 40% trichloroacetic acid solution (*w*/*v*) and lowering their temperature during the subsequent centrifugation (15,000× *g* at 4 °C for 10 min). Five hundred milliliters of soluble fraction was subjected to ultrafiltration in a Millipore stirred cell (Model 8050, Millipore corporation, Burlington, MA, USA) equipped with a 10 kDa nominal molecular mass cutoff polyethersulfone membrane (Trisep Flat Sheet Membrane UF10, Sterlitech Corporation, Auburn, WA, USA), and the retentate, containing melanoidins, was diafiltrated twice with milli-Q water. The melanoidins (the retained fraction) were freeze-dried (FreeZone 12 L Console Freeze Dry System with drying chamber, Labconco, Kansas City, MO, USA).

### 2.3. Simulated In Vitro Gastrointestinal Digestion

In order to obtain bioaccessible samples, the extracted melanoidins were subjected to in vitro gastrointestinal digestion according to the protocol described previously [22], and two digested fractions were obtained: gastric (G) and intestinal (I). An initial oral phase was performed by incubating 500 mg of melanoidins in 10 mL of a simulated salivary fluid (KCl 15.1 mM, KH_2_PO_4_ 3.7 mM, NaHCO_3_ mM, MgCl_2_ 0.15 mM, (NH_4_)_2_CO_3_ 0.06 mM, pH 7) containing α-amylase (75 U/mL) for 2 min at 37 °C. Then, the simulated gastric phase was achieved by adjusting the pH to 1.5 with 1 M HCl and adding 1000 U/mL pepsin solution dissolved in simulated gastric fluid (KCl 6.9 mM, KH_2_PO_4_ 0.9 mM, NaHCO_3_ 25 mM, NaCl 47.2, MgCl_2_ 0.1 mM, (NH_4_)_2_CO_3_ 0.5 mM, pH 1.5) in order to achieve a final pepsin concentration of 500 U/mL. This simulated gastric phase was incubated for 2 h at 37 °C in an orbital shaker (100 rpm). At that point, in order to perform the intestinal phase, simulated intestinal fluid (KCl 6.8 mM, KH_2_PO_4_ 0.8 mM, NaHCO_3_ 85 mM, NaCl 38.4, MgCl_2_ 0.3 mM, pH 7.5) was added, containing 255 U/mL pancreatin and 58.3 mg of bile salts to achieve a final concentration in the sample of 100 U/mL pancreatin and 10 mM bile salts. The intestinal digestion was carried out for 2 h at 37 °C in a thermostatic orbital shaker (100 rpm). The resultant gastrointestinal digested solution was centrifuged (5300× *g*, 10 min), and the soluble portion (supernatant) was considered as the bioaccessible fraction. Two simulated in vitro gastrointestinal digestions were performed for each sample. In the first, only the oral and gastric phases were performed to obtain the bioaccessible gastric fraction (G). In the second, the three phases were performed to obtain the bioaccessible intestinal fraction (I). A blank without melanoidins was included in each step of the in vitro gastrointestinal digestions.

### 2.4. Anti-Helicobacter pylori Activity Test

The effect of two concentrations (2% and 4%) of common and soft bread melanoidins was tested using the microdilution method against the reference strain (43504) of *H. pylori* obtained from the American Type Culture Collection (ATCC; Manassas, VA, USA). These two concentrations were selected based on the results obtained previously against a wide number of microorganisms [16]. Briefly, the stock culture of *H. pylori* (stored at −70 °C in 20% of glycerol) was cultured on Columbia blood agar supplemented with 5% horse blood under microaerobic conditions (5% O_2_, 5% CO_2_, and 90% N_2_) at 37 °C for 5 days. Then, the culture was grown overnight in Brucella broth with 10% fetal bovine serum (FBS) in the same conditions as the incubations. Once the inoculum was prepared, both melanoidins were partially dissolved in Brucella broth with 10% FBS and *Helicobacter pylori* selective supplement to avoid the growth of the native microbiota presented in the melanoidins in 6-well plates; then, *H. pylori* was added at a final concentration of 6 log CFU/mL and the plates were incubated at 37 °C in microaerobic aerobic and shaking conditions for 24 h and 48 h. *H. pylori* was counted in Columbia blood agar supplemented with 5% horse blood and *Helicobacter pylori* selective supplement after incubation in the same conditions as described above.

### 2.5. Cell Culture and Treatment

Human colon adenocarcinoma Caco-2 (HTB 37, 32 passage number), obtained from the American Type Culture Collection (ATCC), were cultured in Eagle’s minimum essential medium (MEM) supplemented with 20% (*v*/*v*) heat-inactivated fetal bovine serum, 1% (*v*/*v*) non-essential amino acids and 1% (*v*/*v*) L-glutamine. The cells were seeded in standard 96-well plates at an initial density of 100,000 cells/mL for 24 h and incubated in a 90% humidity atmosphere with 5% CO_2_ at 37 °C. Then, the cells were exposed for 24 h to 100 or 200 μg/mL of the gastric or intestinal melanoidin fractions. In previous studies, we demonstrated that the incubation of Caco-2 cells with common and soft melanoidins did not affect cell viability [12].

### 2.6. Caco-2 Infection by Helicobacter pylori

The adhesion of *H. pylori* was analyzed by using a method previously described [23]. Briefly, *H. pylori* was grown from the stock as described above and then grown in Brucella broth with 10% FBS for 5 days prior to infection of Caco-2 human epithelial cells. For the infection, *H. pylori* was added to the Caco-2 cells at a multiplicity of infection (MOI) of 25:1 for 6 h. The Caco-2 cells were pretreated with the different concentrations of melanoidins for 24 h and then infected with *H. pylori* for 6 h and incubated at 37 °C under a 5% CO_2_ atmosphere. Next, the *H. pylori* adhesion was measured by a fluorescence assay, and inflammatory (IL-6, IL-8, and IL-10) and oxidative stress (GSH/GSSG and ROS) were also analyzed.

#### 2.6.1. *Helicobacter pylori* Adhesion (Fluorescence Assay)

After 6 h of *H. pylori* exposure to Caco-2 cells, the non-adherent *H. pylori* cells were carefully removed by washing twice for 5 min with 200 µL of tempered PBS 1X, and the Caco-2 monolayer was fixed with 200 µL of 10% p-formaldehyde for 1 h at 4 °C. After three 10 min washes with 200 µL of PBS 1X, 70 µL of rabbit anti-*H. pylori* antibody (Abcam, Cambridge, UK) in PBS 1X (1:30) was added to each well for 24 h incubation at room temperature in darkness. After three 10 min washes with 200 µL of 1% bovine serum albumin in PBS, 70 µL of goat anti-rabbit immunoglobulin G (Alexa Fluor 488; Abcam, Cambridge, UK) in PBS (1:200) was added to each well, and the plates were incubated for 24 h at 37 °C in darkness. Finally, each well was washed four times (10 min per wash) with PBS and the fluorescence was then measured with a multimode microplate reader (Varioskan LUX, Thermo Fisher Scientific, Waltham, MA, USA) by using excitation and emission wavelengths of 485 nm and 535 nm, respectively. The results were expressed as fluorescence intensity.

#### 2.6.2. Measurement of Interleukins

The levels of interleukin-6 (IL-6), interleukin-8 (IL-8), and interleukin-10 (IL-10) in the cell culture supernatants were analyzed with enzyme-linked immunosorbent assay (ELISA) kits, according to the manufacturer’s instructions (Cusabio Technology LLC, Houston, TX, USA).

#### 2.6.3. Determination of Intracellular Reactive Oxygen Species (ROS)

Intracellular ROS production in Caco-2 cells was measured by a 2′,7′-dichlorofluorescein (DCF) assay, with some modifications. Following the treatment and incubation with *H. pylori*, the cells were washed with an external medium (145 mm NaCl, 5 mM KCl, 1 mm MgCl_2_, and 10 mM HEPES). Then, 100 µL of 20 µM DHCF-DA solution in external medium was added to the cells, and basal fluorescence (t0) was measured with a multimode microplate reader (Varioskan LUX, Thermo Fisher Scientific, Waltham, MA, USA) by using excitation and emission wavelengths of 480 nm and 530 nm, respectively. Fluorescence emission was also recorded after incubation in the dark at 37 °C for 120 min (t120), and the variations in the relative fluorescence units (*t*120–*t*0) were calculated for each sample. The results were expressed as a percentage of fluorescence with respect to the control cells incubated with *H. pylori* and not treated with the fractions.

#### 2.6.4. Determination of Reduced/Oxidized Glutathione (GSH/GSSG)

After 6 h of incubation with *H. pylori*, some of the wells were used to measure the GSH/GSSG levels. The Caco-2 cells were trypsinized and suspended in PBS. The Caco-2 suspensions were immediately acidified with 2% of PCA and centrifuged (6500× *g*, 5 min, 4 °C), and the GSH and GSSG levels were determined in the supernatants using Cayman’s GSH assay kit (Cayman Chemical, Co., Ann Arbor, MI, USA). This kinetic spectrophotometric assay evaluates total GSH (reduced + oxidized) by measuring absorbance at 410 nm at 2 min intervals for 20 min. Quantification of GSSG was performed following GSH derivatization with 2-vinylpyridine, and GSH was estimated by subtracting GSSG from the total GSH. All these assays followed the manufacturer’s instructions. The results were finally expressed as a GSH/GSSG ratio.

#### 2.6.5. Immunofluorescence Microscopy

The Caco-2 cells were seeded at a density of 250,000 cells per well in six-well multi-dishes containing glass coverslips and were allowed to reach confluence. Then, the cell monolayers were pretreated with the different concentrations of melanoidins for 24 h; they were then infected with *H. pylori* for 6 h and incubated at 37 °C under a 5% CO_2_ atmosphere. After fixing the cells with 10% p-formaldehyde for 1 h at 4 °C, rabbit anti-*H. pylori* antibody (Abcam, Cambridge, UK) in PBS 1X (1:30) was added to each well for 24 h incubation at room temperature in darkness. After three washes with 1% bovine serum albumin in PBS, goat anti-rabbit immunoglobulin G (Alexa Fluor 488; Abcam, Cambridge, UK) in PBS (1:200) was added to each well, and the plates were incubated for 24 h at 37 °C in darkness. Finally, the cells were mounted with a Fluoroshield mounting medium (Abcam). Images were obtained using a Leica CTR6000 microscope and LAS AF software Version 4.0.0.11706 (Leica Microsystems, Wetzlar, Germany). The image analysis was realized using ImageJ software 1.54f.

### 2.7. Statistical Analysis

The results are presented as a mean ± standard deviation of the independent experiments (*n* = 6). To perform the statistical analysis, the Statgraphics Centurion XVII-X64 software V17.2.07 was used. The results were compared by one-way analysis of variance (ANOVA) followed by a post hoc Student’s *t*-test. The statistically significant differences were determined considering *p* values ranging from 0.05 to 0.001.

## 3. Results and Discussion

High-molecular-weight melanoidins (>10 kDa) were extracted from the crusts of two types of bakery products (common and soft bread), and they were separated from low-molecular components using an ultrafiltration technique. The main characteristics of the melanoidins isolated from common and soft crust bread and the yield, spectrophotometric characteristics, colorimetric properties, antioxidant capacity, cytotoxicity, elemental analysis, volatile compounds, and antimicrobial properties were shown in previous studies [12,16]. In view of the transformations that would take place in bioactive compounds following ingestion, a simulated gastric and intestinal digestion of melanoidins was performed, obtaining two fractions that were used as cell treatments. It is important to understand that although simulated digestion provides an approximation of the processes that occur in the human gastrointestinal tract, in vitro digestion models do not perfectly mimic physiological conditions. For example, static models do not accurately represent enzyme/substrate ratios, transit times, and the spatial and temporal elimination of digested products. Furthermore, the correct separation of released products from undigested components is critical, and a centrifugation step is not sufficient because some undigested substances may form colloidal dispersions. In addition, static in vitro models are not adequate for assessing macronutrient digestibility, due to the fact that pancreatic digestion remains incomplete and requires further breakdown by brush border enzymes [21]. Moreover, product inhibition can occur in the static models. Despite these limitations, the methodology used in this research follows widely accepted standards for the composition and properties of digestive fluids, making it a reliable approach that has been used by several research groups for different purposes [24,25,26].

### 3.1. Bacteriostatic and Bactericidal Effect Against Helicobacter pylori

In previous studies, our research group demonstrated the antimicrobial activity of soft and common bread melanoidins [16]. However, their anti-*Helicobacter pylori* activity has not previously been studied. For that purpose, the bactericidal effects of common (CB) and soft (SB) bread melanoidins at concentrations of 2% and 4% in 24 h and 48 h *H. pylori* cultures were examined.

**Figure 1:**
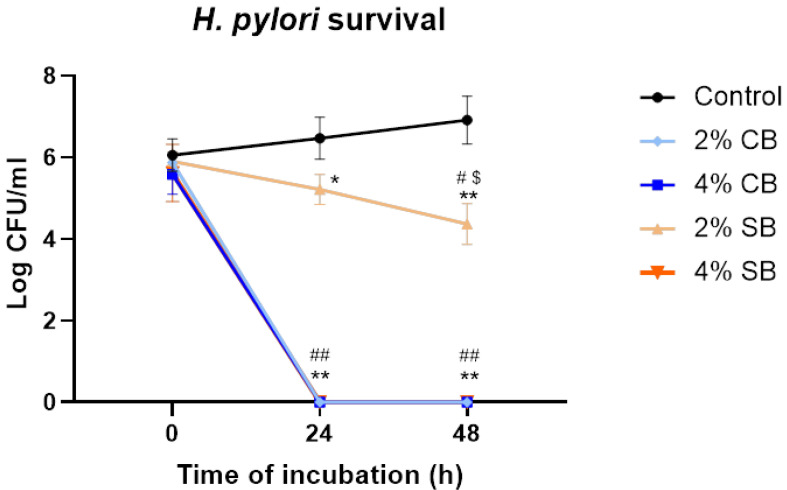
Anti-*H. pylori* effect of different concentrations of common bread (CB) and soft bread (SB) melanoidins. Each point of the graph represents the mean of six replicates derived from three independent experiments, with their respective standard deviation. Comparisons of the different treatments against the control were made by two-way ANOVA followed by Dunnett’s multiple comparisons test, whereas two-way ANOVA followed by Tukey’s multiple comparisons test was used to compare *H. pylori* growth with time for each treatment. Significant differences against the control in the same time period are indicated by * (*p* < 0.01) and ** (*p* < 0.0001). ^#^ (*p* < 0.01) and ^##^ (*p* < 0.0001) indicate differences against time 0 h of each treatment, and ^$^ (*p* < 0.01) is against time of 24 h.

The results show that the melanoidins of CB have a higher bactericidal effect against *H. pylori* (Figure 1); this was already self-evident at 24 h and was maintained at 48 h, at both concentrations tested. A similar result was observed with SB melanoidins at 4%, which had a bactericidal effect at 24 and 48 h. However, the drop observed in bacterial count when the microorganism was incubated with concentrations of 2% was lower compared with the effect at 4%. Moreover, the reduction in bacterial counts at 48 h for the SB melanoidins tested at 2% was significantly higher (*p* > 0.05) than at 24 h (Figure 1). These results showed a bactericidal effect of CB melanoidins at both concentrations evaluated, and also a bactericidal effect at 4% of SB melanoidins and a bacteriostatic effect at 2% of SB melanoidins. Other authors have also observed antimicrobial activity of melanoidins against Gram-negative bacteria, such as *Salmonella* spp., *Campylobacter jejuni*, and *Pseudomonas putida*; however, the reductions obtained were not as high as those obtained in this study [16,27].

### 3.2. Bioaccessible Melanoidins Reduced Helicobacter pylori Adhesion to Caco-2 Cells

The *H. pylori* inhibition by food components, such as products of the Maillard reaction, has been observed in several studies [19,28]. However, there are no studies about the effect of bioaccessible melanoidins on *H. pylori* infection. In this regard, gastrointestinal digestion of melanoidins can release several compounds, which can exert several biological activities [12,29]. Therefore, this study evaluated the bioaccessible fractions of melanoidins from common and soft bread obtained through gastric (G) and intestinal (I) digestion, as this approach allowed their bioactivity against *H. pylori* infection to be distinguished.

**Figure 2:**
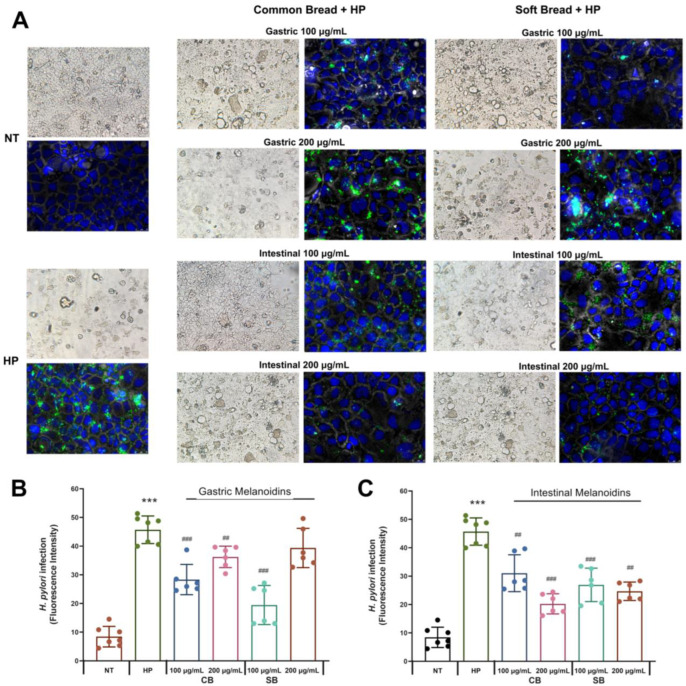
Bioaccessible bread melanoidins reduce *H. pylori* invasion of Caco-2 cells. (**A**) Effect of bioaccessible melanoidins from common and soft bread (200×). *H. pylori* was assessed using antibodies (Alexa Fluor 488, green), nuclear staining was assessed using DAPI (blue), and cell morphology was assessed by phase contrast microscopy (grey). (**B**) Fluorescence intensity of *H. pylori* in Caco-2 cells of non-infected cells (NT), cells infected with *H. pylori* (HP), and cells infected with *H. pylori* and incubated with the gastric melanoidin fractions with 100 or 200 μg/mL. (**C**) Fluorescence intensity of *H. pylori* in Caco-2 cells of cells without treatment (NT), cells infected with *H. pylori* (HP), and cells infected with *H. pylori* and incubated with the intestinal melanoidin fractions with 100 or 200 μg/mL. All values were expressed as mean ± S.D. (*n* = 6 in each group). *** *p* < 0.001 vs. non-infected cells (NT); ^##^ *p* < 0.01, ^###^ *p* < 0.001 vs. cells infected with *H. pylori* (HP). CB: common bread; SB: soft bread; NT: non-infected cells; HP: cells infected with *H. pylori*.

The ability of melanoidins from common and soft bread to interfere with the adhesion of *H. pylori* to Caco-2 cells was evaluated by immunofluorescence microscopy (Figure 2A) and a fluorescence assay (Figure 2B,C) after incubation with 100 or 200 μg/mL of the digestion fractions (G, gastric, and I, intestinal). The Caco-2 model is a well-established in vitro system that is widely used for studying epithelial permeability and the gastrointestinal adhesion of *H. pylori* [30,31]. Although Caco-2 cells are not strictly equivalent to gastric cell lines, they have previously been employed to investigate IL-8 release, *H. pylori*–host interactions, increased permeability, and *H. pylori* translocation [32,33]. Several studies have demonstrated that *H. pylori* not only infects the gastric epithelium but can also affect the proximal small intestine, contributing to the development of duodenal ulcers. Additionally, some reports indicate that *H. pylori* may be associated with both duodenal and colonic mucosa [32,34,35]. Salas-Jara et al. (2016) [36] demonstrated that the larger surface area of Caco-2 cells enables *H. pylori* to form a more extensive biofilm compared to gastric cells. Furthermore, *H. pylori* disrupts the normal duodenal microbiota, a phenomenon linked to the development of diseases associated with *H. pylori* infection [37,38].

The melanoidins of both bread crusts at a concentration of 100 μg/mL inhibited the adhesion of *H. pylori* up to 30%. At this concentration, the SB fractions were more effective than CB fractions, and the gastric fraction of SB was the most effective with 57.4% inhibition. At a concentration of 200 μg/mL, the soft bread gastric fraction did not have any effect on *H. pylori* adhesion (*p* < 0.05, Student’s *t*-test) (Figure 2B), whereas the other fractions were effective. A maximal inhibitory effect at this concentration was observed with the intestinal fraction of common bread, which decreased the levels of *H. pylori* adhesion by 55.7%.

Our studies show that both types of melanoidins decreased the levels of *H. pylori* adhesion to intestinal cells, but their degree of inhibition depended on their digestion (gastric or intestinal) and concentration, probably as a response to the difference in the bioactive compound’s composition in each fraction [20]. The gastric fraction at the concentration of 100 μg/mL exerted a higher inhibition of *H. pylori*, but the intestinal fractions showed a more pronounced effect at 200 μg/mL. Moreover, the two types of melanoidins had a different behavior, whereby the soft bread gastric fraction (100 μg/mL) was the most effective, and for the common bread, it was the intestinal fraction (200 μg/mL) (Figure 2). In this regard, other studies also showed that the antibacterial activity of melanoidins is affected by the substrates used in the Maillard reaction to produce them [27], and this could reflect the diversity observed between melanoidins derived from different types of bread. Additionally, there are some results that are important to highlight when considering their relevance in the pathophysiology of *H. pylori*. Soft bread melanoidins showed a higher inhibition for the gastric fraction, and this result is of great importance considering that the gastric mucosa is the most critical site in the *H. pylori* infection. Furthermore, the inhibition of *H. pylori* adhesion in both gastric melanoidins was higher for the lower dose (Figure 2B).

Some studies indicate that MR intermediates such as melanoidins show inhibition of the growth of *H. pylori* due to the inhibition of the binding of urease to mucin [19]. However, it must be underlined that Caco-2 cells are not able to produce mucus, which is crucial in the study of the anti-urease effect. Thus, the inhibitory effect observed in our study probably responds to another mechanism of action of the melanoidins against *H. pylori* adhesion that is more related to the melanoidins’ antioxidant capacity [20]. Membrane integrity is a crucial component of epithelial barrier function and could be affected by oxidative stress and microbial infection. In this regard, melanoidins are bioactive compounds that can modulate the cellular redox state involved in the inflammatory response, together with the enhancement of cellular junction proteins through the regulation of redox-sensitive factors [12,39]. This regulatory and protective effect in the epithelium, together with the potential perturbation of the bacterial integrity could explain the *H. pylori* inhibition by melanoidins [20,27].

### 3.3. Bioaccessible Melanoidins Reduced Inflammation of Gastric Epithelium

The Caco-2 cell line is a well-established model of the gastrointestinal epithelium that is capable of differentiation and polarization, and it releases several inflammatory mediators because of *H. pylori* infection [40]. The effects of common and soft bread melanoidins on *H. pylori*-induced inflammation were evaluated by measuring IL-6, IL-8, and IL-10 levels.

**Figure 3:**
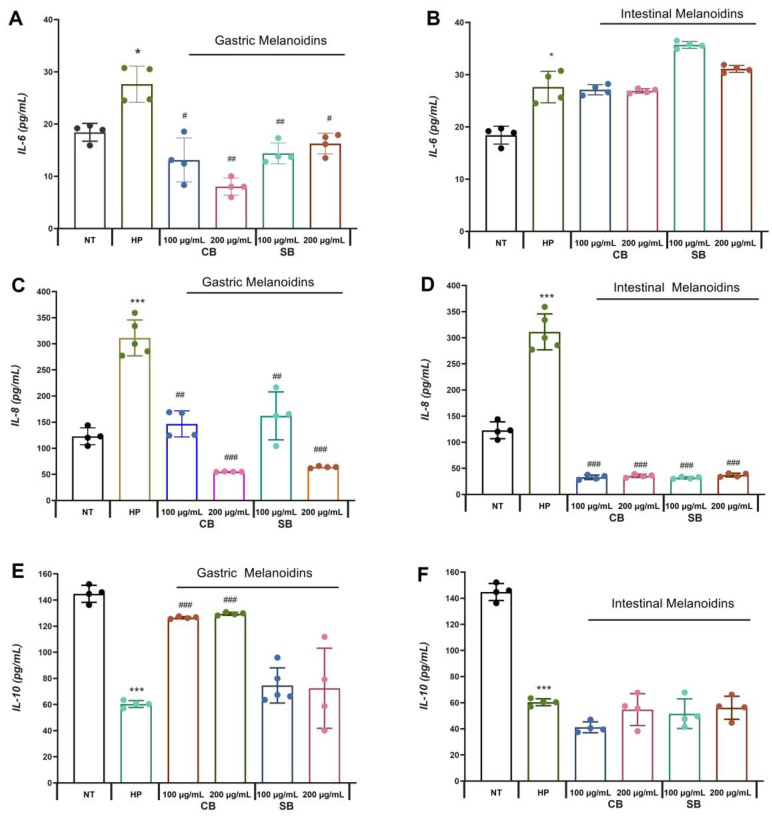
Bioaccesible bread melanoidins attenuate inflammation in Caco-2 cells. (**A**,**B**) Interleukin 6 (IL-6) levels. (**C**,**D**) Interleukin 8 (IL-8) levels. (**E**,**F**) Interleukin 10 (IL-10) levels. All values were expressed as mean ± S.D. (*n* = 4 in each group). * *p* < 0.05 *** *p* < 0.001 vs. non-infected cells (NT); ^#^ *p* < 0.05; ^##^ *p* < 0.01, ^###^ *p* < 0.001 vs. cells infected with *H. pylori* (HP). CB: common bread; SB: soft bread; NT: non-infected cells; HP: cells infected with *H. pylori*.

Significative increases in the levels of IL-6 and IL-8 release from Caco-2 were detectable by ELISA after incubation with *H. pylori* (HP) compared with the control group (NT) (Figure 3). These results are due to the interaction of *H. pylori* with the gastric epithelium that promotes an inflammatory response which is mediated by pro-inflammatory cytokines, such as IL-6 and IL-8.

It is known that IL-6 is involved in the defense of the organism, functioning as a messenger between an innate and adaptive immune response, but the presence of *H. pylori* promotes an increase in the synthesis of IL-6, causing inflammation and gastritis [3]. In our study, it was observed that the pretreatment with gastric fractions of both common and soft bread melanoidins reduced the levels of IL-6 from the infected cells (Figure 3A), whereas the intestinal fractions did not have a significative effect (Figure 3B). These results are consistent with those presented in a report by other authors [41], who showed a reduction in IL-6 release in intestinal cells by digested melanoidins from coffee, where the digestion process had significant relevance in the release of molecular compounds with high anti-inflammatory action. Some authors indicate that the anti-inflammatory effects observed for the melanoidin treatment are mediated, at least in part, by the antioxidant mechanisms of ROS scavenging [1]. The involvement of the NF-κB oxidative pathway as a modulator of oxidative stress is commonly associated with the regulation of the inflammatory mediators’ release and the oxidative stress increase in intestinal cells as a consequence of microbial infection [42]. Several studies have shown the melanoidins’ effect on the transcription factors nuclear factor erythroid 2-related factor 2 (Nrf2) and NF-kB [43]. In addition, NF-κB and Nrf2 crosstalk also have a relevant involvement in the regulation of IL-6 in response to many microbial infections, where Nrf2 disruption leads to the increase in pro-inflammatory cytokines and the decrease in anti-inflammatory ones [44]. Thus, the observed effects of bread melanoidins probably respond to a regulation of one or both transcriptional factors and need to be more studied.

In contrast, gastric and intestinal melanoidin fractions reduced the pro-inflammatory cytokine IL-8 release from *H. pylori*-infected cells from 47.9% to 89.8% (Figure 3C,D). IL-8 has been identified as a key mediator of epithelial immune responses affecting the activation and migration of immune cells (neutrophils, basophils, and T cells) [8,45]. Therefore, the modulation of the expression of IL-8 by intestinal epithelial cells may have a therapeutic benefit. Some studies indicate that *H. pylori* can induce the expression of IL-8 activated by the oxidative pathway NF-κB in gastric epithelial cells [6,7]. This pro-inflammatory cytokine is overexpressed in the gastric mucosa exposed to *H. pylori*, and consequently, the expression of IL-8 plays an important role in gastric cancer. In accordance with the results, several antioxidant compounds are capable of inhibiting *H. pylori*-induced IL-8 [46]. Furthermore, an anti-inflammatory effect of melanoidins was reported in Caco-2 cells induced by gamma interferon and phorbol ester by the ability to inhibit nitric oxide (NO) and IL-8 production [47]. Our results are also in accordance with those presented by other authors [48], who showed that low concentrations (100 μg/mL) of melanoidins exhibited anti-inflammatory effects.

On the other hand, *H. pylori* infection reduced IL-10 release by 58.3%. Pretreatment with gastric fractions of common bread melanoidins enhanced the release of IL-10 from infected cells (Figure 3E). This anti-inflammatory effect was more extensive for the gastric fractions, which reduced both IL-6 and IL-8 release from infected cells, and increased IL-10 release. IL-10 is an anti-inflammatory cytokine released by the gastric epithelial cells in an *H. pylori* infection. Gastric inflammation produced by *H. pylori* invasion shows a reduction in the immune response and lower production of IL-10 associated with a downregulation of the FOXP3 gene [49]. In accordance with the results, some authors observed an enhanced IL-10 release in Caco-2 cells treated with digested melanoidin-rich foods [41]. Our results are in disagreement with those observed by some authors [50], who reported a slight impact of fruit MR products on the IL-10 production. The structural properties of melanoidin compounds are largely unknown and depend on the source of melanoidins, but they are well recognized for their diversity and heterogeneous structure [51]. Melanoidins from the crust of common bread may have bioactivity predominantly in the gastric fraction and could explain the better release of IL-10 by this fraction [20].

Considering that intestinal epithelial cells may affect the local immune system with their cytokine secretion and thus promote an inflammatory response, the modulation of cytokine release by the digested melanoidins is a potential benefit in *H. pylori* infection, mainly in the gastric mucosa.

### 3.4. Bioaccessible Melanoidins Alleviated Oxidative Stress by Reduction in ROS Levels and Improvement of Antioxidant Defenses

**Figure 4:**
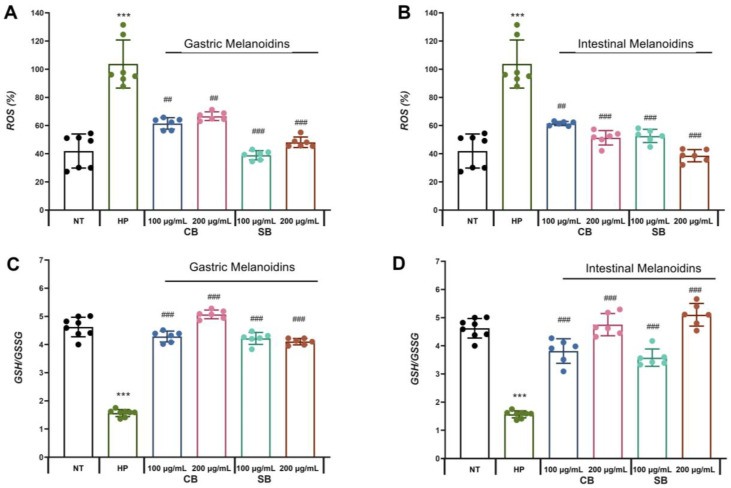
Bioaccessible bread melanoidins alleviated the level of intracellular oxidative stress. (**A**,**B**) Reactive oxygen species (ROS) levels in Caco-2 cells. (**C**,**D**) Cellular redox status represented with reduced glutathione (GSH)/glutathione disulfide (GSSG) ratio in Caco-2 cells. All values were expressed as mean ± S.D. (*n* = 6 in each group). *** *p* < 0.001 vs. non-infected cells (NT); ^##^ *p* < 0.01, ^###^ *p* < 0.001 vs. cells infected with *H. pylori* (HP). CB: common bread; SB: soft bread; NT: non-infected cells; HP: cells infected with *H. pylori*.

The induction of oxidative stress in the infected cells has been described as one of the *H. pylori* virulence factors, which are responsible for inducing the pro-inflammatory state in epithelial cells [10]. In our study, it can be observed that *H. pylori* significantly increased the levels of ROS in the Caco-2 cells by more than 50% (Figure 4A,B). These results are consistent with other studies that showed the production of ROS by *H. pylori* itself and ROS accumulation in gastric epithelial cells, suggesting that the increase in oxidative stress is an important mechanism leading to gastrointestinal epithelial injury [50]. According to previous studies [2], one of the virulence mechanisms of *H. pylori* is mediated by the enzyme γ-glutamyl-transpeptidase that promotes the release of hydrogen peroxide, inhibiting cell proliferation and inducing apoptosis and necrosis of the gastric epithelial cells and increased secretion of IL-8 through NF-κB activation. The presence of melanoidins in the incubation medium of Caco-2 infected with *H. pylori* showed an effect that is dependent on the source of melanoidins. All types of bioaccessible soft bread melanoidins tested reduced the ROS levels significantly but some of the fractions had a more marked effect. Gastric melanoidins from soft bread allowed a reduction of more than 50% of ROS in the Caco-2 cells infected with *H. pylori*, while gastric melanoidins from common bread (CB) caused a reduction of between 35.7% and 40.7% (Figure 4A). In the case of intestinal melanoidins, all the fractions showed a significant reduction in ROS levels of between 40.7% and 60.8%. Interestingly, the intestinal fractions showed the greatest reduction in IL-8 levels. In addition, the reduction in ROS levels by the intestinal fractions showed a dose-dependent effect, with the highest reductions at 200 µg/mL being approximately 50.5% and 60.8% (Figure 4C).

Another molecule involved in the modulation of the redox cell state is glutathione, which is the most important endogenous non-enzymatic mechanism protecting cells against oxidative damage and was selected as a marker of the overall cell redox environment. *H. pylori* significantly reduced the rate of reduced glutathione (GSH)/glutathione disulfide (GSSG) ratio (Figure 4C,D), supporting the induction of oxidative stress in Caco-2 cells during *H. pylori* infection. These results are in accordance with other studies that revealed a reduction in the GSH levels during *H. pylori* infection in the infected cell. Furthermore, other studies also suggested that the depletion in GSH in the gastric mucosa due to *H. pylori* infection produces a more sensitive mucosa that is susceptible to oxidative damage and the effects of ROS involved in their carcinogenic effect [52]. This decrease in GSH levels facilitates the colonization of the gastric mucosa by *H. pylori*. This is consistent with our results, which showed the highest infection in the non-treated *H. pylori*-infected (HP) Caco-2 cells (Figure 2) that had the lower GSH/GSSG levels (Figure 4C,D). In contrast, when *H. pylori*-infected epithelial cells were pretreated with common or soft bread melanoidin fractions, an increase in the GSH/GSSG ratio was observed. The highest levels of GSH/GSSG were observed in the intestinal fraction of the soft bread melanoidins at 200 µg/mL, with a 3.25-fold increase (Figure 4D). These results are consistent with the lowest levels of ROS (Figure 4B). This increase in the glutathione levels by the treatments improved the redox status, and they reached those of the uninfected cells. The antioxidant capacity of melanoidins may be responsible for their antioxidant potential and the improvement of the redox cellular status.

The mechanisms by which melanoidins may act as antioxidants include reactive oxygen scavenging, reducing agents and metal chelating agents, modulation of redox signaling pathways, and improvement of cell antioxidant systems [53]. In accordance with some authors that suggested the existence of positive correlations between antioxidant activity and melanoidin color [54], the highest color of soft bread melanoidin [12] could explain the highest antioxidant protection of the soft bread fractions.

## 4. Conclusions

In summary, bioaccessible melanoidins from the crust of common bread and soft bread showed different cellular responses to the *H. pylori* infection of Caco-2 cells. The results suggest that the soft bread melanoidins, with the highest antioxidant capacity, are the most effective in the prevention of *H. pylori* infection by its bactericidal effect, reducing the bacterial invasion of Caco-2 and both inflammation and oxidative stress in epithelial cells. Moreover, in view of the relevance of the gastric mucosa as the main site of action of *H. pylori*, the most effective action of the gastric fractions against invasion, inflammation, and oxidative stress, can be stressed as an advantage. Additionally, the better reaction of the gastric soft melanoidins at the lower concentration may be considered as a benefit considering the importance of reducing the amount of melanoidins added into food and reducing the potential alteration of organoleptic properties.

## Data Availability

The original contributions presented in this study are included in the article. Further inquiries can be directed to the corresponding author.

## Abbreviations

The following abbreviations are used in this manuscript:

CB	Common bread melanoidins
DCFH-DA	2′,7′-dichlorofluorescein diacetate
FBS	Fetal bovine serum
G	Gastric bioaccessible fraction
GTT	γ-glutamyl-transpeptidase
GSH/GSSG	Reduced/oxidized glutathione
HP	*Helicobacter pylori*
I	Intestinal bioaccessible fraction
IL-8	Interleukin 8
IL-6	Interleukin 6
IL-10	Interleukin 10
MEM	Eagle’s minimum essential medium
MOI	Multiplicity of infection
MR	Maillard reaction
NT	Cells without treatment and non-infected
ROS	Reactive oxygen species
SB	Soft bread melanoidins
